# Validation of the Chinese version stage of recovery instrument-30 (STORI-30) for adults with severe mental illness

**DOI:** 10.1186/s12888-023-04954-y

**Published:** 2023-07-04

**Authors:** Sharon Wing-Yung Lau, Colin Kwok-Man Law, Siu-Man Ng

**Affiliations:** 1 Occupational Therapy Department, Castle Peak Hospital, Hospital Authority, Hong Kong, Hong Kong; 2 Department of Social Work and Social Administration, University of Hong Kong, Hong Kong, Hong Kong

**Keywords:** Stage of recovery, Mental health recovery, Recovery measurement, Severe mental illness

## Abstract

**Background:**

Stage of Recovery Instrument-30 (STORI-30) is grounded in a five-stage model of psychological recovery, and serves as measuring recovery stage of people with mental illness.

**Aims:**

To develop and validate the Chinese version STORI-30 on adults with severe mental illness.

**Methods:**

STORI-30 was translated to traditional Chinese through forward-backward method. An expert panel and potential users evaluated face validity and content validity. The Chinese version STORI-30 plus other convergent and divergent scales were then administered to 113 participants for field test.

**Results:**

Face and content validity were confirmed with acceptable Content Validity Index and high inter-rater agreement. Exploratory factor analysis revealed a three-factor structure. An ordinal sequence was presented among the five subscales, similar to the original version. Construct validity was supported by positive correlations with recovery and mental well-being scales, and negative correlation with self-stigma scale. Good internal consistency (Cronbach’s α = 0.78–0.86) and high level of test-retest reliability (Intraclass correlation coefficient = 0.96) were obtained.

**Conclusions:**

Chinese STORI-30 presents satisfactory psychometric properties in internal consistency, construct convergent and divergent validity, and test-retest reliability. The three-factor structure revealed does not echo the original five-stage recovery model. Further studies exploring the underlying structure are warranted.

## Introduction

People with severe mental illness (SMI) experience functional and psychosocial impairments which noticeably limit major life activities [1, 2]. Since the recovery movement in 1980s, there was a shift from long-established medical approach to a recovery model among mental health systems [3]. Service providers aimed at boosting psychological recovery in the healing progress, rather than emphasising symptom management. Consumers defined psychological recovery as “the establishment of a fulfilling life and a positive self-identity founded on sense of hope and self-determination” [4–6]. It is imperative to facilitate individuals not just to restore premorbid functioning but to live a purposeful life in conjunction with achieving personal values [7].

Andresen et al. [8] advanced a consumer-oriented stage model of psychological recovery, which set forth a framework consisting four recovery dimensions: finding and maintaining hope; re-establishing a positive identity; finding meaning of life; and taking responsibility for wellbeing. Moreover, a five-stage recovery model was proposed. Each stage is characterised by a combination of varied accomplishment in the four dimensions of recovery. The five stages are briefly:

1. Moratorium, a time of self-protective withdrawal, denial of illness identity, with a profound sense of hopelessness.
2. Awareness, a turning point in recovery, with a glimmer of hope of a better life and aware a possible self rather than illness identity.
3. Preparation, a stage of groundwork by exploring introspective values and external resources, so as to set autonomous goals.
4. Rebuilding, a phase of hard work involves taking responsibility for managing illness and taking control of own life.
5. Growth, an ongoing phase of striving for personal growth and signifying psychological well-being.

Under evidence-based practice of mental health services, only if clinicians assess consumers’ personal stage of recovery can the clinicians deliver stage-specific interventions and monitor recovery progress. Grounding in this stage model, Andresen et al. [9, 10] developed the Stages of Recovery Instrument (STORI) and a corresponding short form, STORI-30, in order to assess consumer-defined stage of recovery. In the past decades, Hong Kong mental health workers validated numerous assessments about recovery components or factors [11]. Recovery Assessment Scale - Chinese version (RAS-C) and Mental Health Recovery Measure (MHRM) are two popular measurements utilised in clinical practice and research studies. RAS-C is designed to measure five factors of psychological recovery but it does not tap on recovery stage context [12]. MHRM is rooted in a three-phases recovery model [13]. But the scale scoring indicates neither which recovery phase the client is situated in, nor the chronological relationship of the three recovery phases.

This study chose STORI-30 for validation because it is consumer-oriented, easy to administer in clinical settings, and has gone through proper development process. Most importantly, its substantial feature in determining recovery stages is not achieved by any other instrument [14]. Taking note of multidimensional and dynamic characteristics of recovery stages, clinicians shall identify and deliver specific services regarding the stage wherein the consumer is positioned [15]. In addition, mental health services are expected to demonstrate true effect of boosting people in recovery with measurable indicators [16]. In fact, STORI-30 caught attention in evaluation of clinical treatment and training. It was applied as an outcome measurement in recovery decision-making studies for people with SMI [17, 18].

Nevertheless, it is necessary to address the uncertainty in the factor structure of STORI-30. The development study of STORI-30 revealed only four factors rather than the expected five factors by exploratory factor analysis (EFA). On the other hand, the development of STORI and its validation study in the United Kingdom came up with three-clusters solution by hierarchical cluster analysis. These findings warranted attention for re-examination of the structural validity. Furthermore, it is noteworthy that both STORI and STORI-30 were developed in Australia, and only validated among western countries [19, 20]. It remains unexplored if the instrument is applicable under local context or not. Han and Chen [21] argued that the emphasis on familism among Chinese culture might result secondary stigma extending from the individual to his or her family, and this could be conductive to hinder psychological recovery. Therefore, it is compulsory to seek content-related evidence on local adults with mental illness.

Research question of present study is designed to rigorously study psychometric properties of the scale: “Is Chinese version STORI-30 a valid and reliable instrument, in terms of face validity, content validity, structural validity, construct convergent and divergent validity, criterion validity, internal consistency and test-retest reliability?”. If the scale is successfully validated on local consumers, it would empirically confirm the five-stage of recovery model in theoretical context, and enrich the conceptualisation in assessment of recovery stage.

## Materials and methods

The first phase of present study was to translate STORI-30 into traditional Chinese, and evaluate its face and content validity using an expert panel review method. Phase two was a pilot study to assess feasibility from consumers’ perspective. The last phase was a field test to gain evidence of other psychometric properties.

### Participants

People with SMI were recruited by convenience sampling from outpatient services of the Hong Kong Hospital Authority and two non-governmental organisations. All participants met the follow inclusion criteria:

1. Aged between 18 and 64 years.
2. Able to understand spoken Cantonese and written traditional Chinese.
3. With diagnosis of schizophrenia, mood disorders and other psychotic disorder according to International Classification of Disease-10 assessed by psychiatrists [22].
4. In stable phase of mental illness as defined by no hospitalisation and no changes in drug regimen in the past 3 months.
5. Able and willing to provide written informed consent for participation in the study.

They were excluded if they were (1) complicated with dementia, mental retardation, serious medical or neurological conditions, or (2) with history of organic brain disorder. A sample size of minimum five participants in each item was preferred for EFA and thus 150 participants were required [23]. Sample size estimation of test-retest reliability was done by Power Analysis & Sample Size software program [24]. Twenty-six participants were required to detect intraclass correlation coefficient (ICC) value of 0.50, while fixing 0.05 alpha, 0.80 power, and allowing 15% non-response rate [25].

### Instruments

#### Stage of recovery instrument-30 (STORI-30)

STORI-30 consists of thirty statements rating on a six-point scale. Recovery stage is determined according to the participant’s highest score among five subscales. If two subscales scores are the same, stage allocation would be judged on the higher stage. It overall demonstrated satisfactory psychometric properties [10]. Strong positive correlations were found between adjacent stages (r = 0.78, p < 0.01 between Stage 3 and 4), while negative correlations were shown between Stage 1 and other subscales (r = -0.51, p < 0.01 between Stage 1 and 5). Internal consistency of each subscale was satisfactory (Cronbach’s α = 0.77 to 0.85). Concurrent validity was established by correlation of recovery stages with Recovery Assessment Scale, which had significant negative correlation with Stage 1 (r = -0.65, p < 0.01) and strong positive correlation with Stage 5 (r = 0.77, p < 0.01).

#### Recovery assessment scale - Chinese version (RAS-C)

RAS-C is a 24-item scale measuring subjective views in recovery. It comprises five factors: “personal confidence and hope”, “willingness to ask for help”, “goal and success orientation”, “reliance on others”, and “no domination by symptoms”. Its psychometric properties were validated among Chinese adults with mental illness in Hong Kong [12]. It presented satisfactory internal consistency among subscales (Cronbach’s α = 0.73 to 0.93). Its concurrent validity was established with Recovery Markers Questionnaire (r = 0.72, p < 0.001), and construct validity was demonstrated with life satisfaction scale (r = 0.62, p < 0.001) and self-stigma scale (r = -0.35, p < 0.001). We anticipated a positive correlation between Chinese STORI-30 stage allocation with RAS-C, because people shall attain better recovery factors at a later recovery stage.

#### Chinese version of the short warwick-edinburgh mental well-being scale (C-SWEMWBS)

C-SWEMWBS consists of seven positively-phrased statements and aims at assessing quality of life among people with mental illness [26]. Its reliability and validity were confirmed including good internal consistency (Cronbach’s α = 0.89) and high test-retest reliability (r = 0.678, p < 0.001). Concurrent validity was demonstrated by positive correlation with the 5-item World Health Organization Well-being Index (r = 0.49, p < 0.001). In this study, we expected mental well-being to be a convergent measure with recovery stages.

#### Self-stigma scale – short form (SSS-S)

SSS-S is a 9-item instrument assessing the extent to which people with mental illness internalise negative stereotypes towards self-identity [27]. The higher score indicates endorsement of self-stigma to a greater extent. Its internal consistency was tested by Cronbach’s α with 0.87. Convergent validity was proven by a moderate correlation between self-stigma and Stigmatization Scale (r = 0.54, p < 0.001). In this study, we hypothesised a negative correlation between Chinese STORI-30 stage allocation with the SSS-S because people positioning at earlier recovery stages shall present higher extent of self stigma.

#### Translation process of Chinese version STORI-30

After translation approval was granted from the colleague of original author Dr. Retta Andresen who had retired, we translated the STORI-30 into traditional Chinese following the forward-backward translation methodology [28]. One of the three translators was an expert in recovery-oriented services and holding a master degree of mental health. Another two translators were both naïve in mental health, but holding a master degree of translation and knowledgeable of English-speaking culture. Translators were all independent from each other during translation process, with the purpose of enabling divergent interpretation of ambiguous terms. Harmonisation across forward and backward translations was an important quality-control step. An expert panel was then recruited which consisted of five bilingual mental health professionals in multidiscipline (associate consultant, senior occupational therapists and advanced practice nurse). Panel members rated on a questionnaire and gave comments related to face and content validity. The revised draft was then administered to ten consumers for pilot study to evaluate feasibility. Necessary adjustments were made to affirm conceptual equivalence and translation appropriateness. They highlighted certain wordings on the scale and emphasised the continuity nature of recovery stages. For instance, they suggested to differentiate explicitly between “I want to start”, “I have just started to” and “I am starting to”. Statements starting with “I want to start” indicated an initial stage that the person was still processing in mind without any action taken. Also, “I have just started to” differed from “I am starting to”, in which the former phrase indicated the statement happened recently but the latter one indicated the action is happening at the current stage and still in progress.

### Procedures

After obtaining written consents from participants, they were asked to complete four self-reported questionnaires (Chinese version STORI-30, RAS-C, C-SWEMWBS, SSS-S) which approximately took forty minutes to complete. Participants were invited for retest by convenience sampling, and their caseworkers were invited to rate participants’ recovery stage as a way to evaluate criterion validity. Concepts of recovery stages and the Chinese version STORI-30 were explained to all caseworkers with supplementary information, so as to ensure their thorough understanding. They rated on the scale based on their clinical judgement and perception on clients’ recovery stages.

### Statistical analysis

Face and content validity was evaluated by Content Validity Index (CVI) in both item- and scale-level [29]. Inter-rater agreement of feasibility questionnaire was examined by percentage of exact and adjacent agreement [30]. Structural validity was assessed by EFA with principal axis factoring. Kaiser criterion of eigenvalues and visual scree plot test were employed to determine number of factors to be extracted [31, 32]. Promax rotation method was chosen because components were allowed to be correlated. A rotated factor matrix was created to confirm the subscales were highly correlated to a specific factor. Dimensionality of each extracted factor was analysed by internal consistency using Cronbach’s coefficient alpha. A value of 0.70 or above was considered a satisfactory level of internal reliability [33]. Correlations between the five subscales were also investigated with Pearson correlations, in order to prove the fundamental structure of the recovery stage framework.

Correlations of Chinese STORI-30 stage allocation with other convergent and divergent instruments were examined by Pearson correlations. For criterion validity, Cohen’s kappa was run to assess agreement between clinicians’ judgement and participants’ subjective recovery stage. Kappa values 0.20–0.40 are regarded as fair, 0.40–0.60 as moderate, 0.60–0.80 as good, and 0.80 above as excellent agreement [34]. Test-retest reliability of stage allocation and individual five subscales were assessed by ICC. Values below 0.50 indicate low level of reliability, values between 0.50 and 0.75 indicate moderate level, values above 0.75 indicate satisfactory level [35].

## Results

### Demographic data

Under the impact of coronavirus disease 2019 and mental health services restriction, only 113 participants were recruited at last. Around half were male (n = 55, 48.7%) and the mean age of all participants was 43.4 years (SD = 12.0). Majority of them (n = 75; 66.4%) were diagnosed with schizophrenia. On average, their duration of mental illness was 16.0 years (SD = 11.4) and 5.2 years from last hospitalisation (SD = 6.6). Table 1 summarises the demographic characteristics of all participants.

**Table 1:** Demographic characteristics of study participants (N = 113)

Variables	Categories	n (%)
Gender	Male	55 (48.7)
	Female	58 (51.3)
Age	20–29	20 (17.7)
	30–39	21 (18.6)
	40–49	28 (24.8)
	50–59	36 (31.9)
	60–64	8 (7.1)
Educational level	Primary or below	16 (14.2)
	Junior secondary	30 (26.5)
	Senior secondary	51 (45.1)
	Tertiary or above	16 (14.2)
Employment status	Unemployed	22 (19.5)
	Open employment	29 (25.7)
	Supported employment	14 (12.4)
	Shelter workshop	26 (23.0)
	Day training	19 (16.8)
	Housekeeper or retired	3 (2.7)
Diagnosis	Schizophrenia	75 (66.4)
	Depression	20 (17.7)
	Bipolar affective disorder	10 (8.8)
	Schizoaffective	6 (5.3)
	Psychosis with history of substance abuse	2 (1.8)
Duration of mental illness (years)	1–10	48 (42.5)
	11–20	22 (19.5)
	21–30	31 (27.4)
	31–40	12 (10.6)
Years since last hospitalisation	Below 1	17 (15.0)
	1–10	75 (66.4)
	11–20	15 (13.3)
	21–30	5 (4.4)
	31 or above	1 (0.9)

### Face and content validity

Item-level CVIs were ranged from 0.80 to 1.00, scale-level CVI/average method was 0.95, and scale-level CVI/universal agreement method was 0.75.

### Feasibility measurement

From the feasibility questionnaire, a 90–100% inter-rater agreement was resulted on the questions related to brevity, simplicity and acceptability. Except the item of relevance, 70% of participants showed agreement but the others revealed that they did not often concern personal recovery in daily life, as long as they could maintain stable mental state with psychiatric medications.

### Structural validity

#### Recovery stage allocations

Over half of participants were allocated to Stage 4 (n = 29; 25.7%) and Stage 5 (n = 41; 36.3%). Only minority were positioned at Stage 1 and 2 (n = 30, 26.5%).

#### Factor structure

Item analysis was first conducted for evaluating the extent to which the item correlates with the sum of other items on the same subscale. “Corrected item-total correlation” were ranged from 0.41 to 0.77. Using Cohen’s rule of thumb, it is best to have 0.37 or above, and results implied no sign of multicollinearity because the values were below 0.80 [36, 37]. Also, removing any item did not lead to a rise of alpha value. Therefore, all thirty items were worthy of retention.

**Fig. 1:**
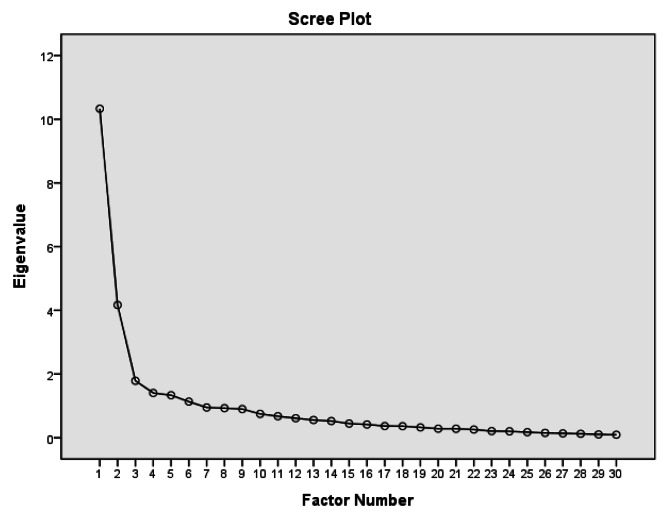
Visual scree plot of Chinese STORI-30

Kaiser-Meyer-Olkin measure was 0.84, which was greater than desired 0.70 [38]. Bartlett’s test of sphericity was found to be significant with a p value < 0.001 (χ2 = 2194.05, df = 435), thus fulfilling prerequisites of EFA. Visual scree plot (Fig. 1) showed that the curve levelled off after the third components, with eigenvalues of the first three factors greater than 1 (9.95, 3.74, and 1.39). These findings suggested a three-factor solution and together the three factors explained total 50.28% of variance. Table 2 displays the pattern matrix from EFA. With significant loading set at 0.40, each factor comprised items as follows:

**Table 2:** Exploratory factor analysis of Chinese STORI-30 (N = 113)

Item	Factor loading		
	Factor 1	Factor 2	Factor 3
28	0.80		
22	0.80		
24	0.78		
23	0.75		
7	0.75		
3	0.63		
12	0.54		
2	0.50		
25	0.47		
29	0.41		
17	0.40		
27	0.40		
20		0.82	
10		0.79	
18		0.77	
13		0.70	
19		0.70	
15		0.68	
14		0.67	
8		0.62	
9		0.61	
30		0.43	
16			0.82
6			0.72
26			0.70
1			0.68
21			0.65
11			0.61
5			*-0.53*
4			*-0.41*
Eigenvalue	9.95	3.74	1.39
Percentage of variance	33.16%	12.48%	4.64%

Kaiser-Meyer-Olkin value = 0.84; Bartlett’s test, X^2^ = 2194.05, df = 435, p < 0.001

- Factor 1: all six items of Stage 2, three items of Stage 3, two items of Stage 4 and one item of Stage 5.
- Factor 2: three items of Stage 3, three items of Stage 4, and four items of Stage 5.
- Factor 3: all six items of Stage 1.

The Cronbach’s alpha coefficients for the three factors were 0.90, 0.90 and 0.85 respectively. Factor 1 was moderately correlated with Factor 2 by 0.61 and negatively correlated with Factor 3 by﻿ 0.19. Factor 2 moderately correlated with Factor 3 by -0.52.

Nevertheless, several problems arise regarding this three-factor solution. First, item 4 and 5 had loading on Factor 3 but in opposite direction with other items. This is because both items are belonged to later recovery stages and conceptually in contrast to other items under Factor 3. Also, eigenvalue of Factor 3 only accounted for an additional 4.64% of the total variance. Small group of participants positioning at Stage 1 might account for this finding.

#### Internal consistency of individual recovery stage

High Cronbach’s coefficient alpha values were resulted of all five subscales: Stage 1 α = 0.85, Stage 2 α = 0.79, Stage 3 α = 0.78, Stage 4 α = 0.80, and Stage 5 α = 0.86. All values were greater than 0.70, indicating high reliability [39].

#### Intercorrelations of recovery stages

A distinct pattern was found in the correlations between the five subscales. There were small to large negative correlations between Stage 1 and the other recovery stages (r = -0.25 to -0.51, p < 0.01) except Stage 2. Adjacent stages showed strong positive correlations, such as Stage 2 and Stage 3 (r = 0.81, p < 0.01), Stage 3 and Stage 4 (r = 0.80, p < 0.01), Stage 4 and Stage 5 (r = 0.83, p < 0.01). Conversely, distant stages showed lower levels of positive correlations, for instance, Stage 2 and Stage 5 (r = 0.40, p < 0.01).

### Construct convergent and divergent validity

Table 3 presents correlations of the three factors with convergent and divergent measures. Factor 1 moderately correlated with recovery and well-being scales (r = 0.34 to 0.56, p < 0.01) and Factor 2 showed stronger correlations (r = 0.65 to 0.76, p < 0.01). Both Factor 1 and 2 showed negative correlations with self-stigma scale. On the other hand, Factor 3 had negative correlations with recovery and well-being scales, but a positive correlation with self-stigma scale (r = 0.49, p < 0.01).

In addition, correlations between stage allocation and the five subscales scoring with other validation scales were shown on Table 4. As expected, stage allocation had positive and significant correlations with RAS-C (r = 0.61, p < 0.01) and C-SWEMWBS (r = 0.60, p < 0.01). A discrete correlation pattern was shown between Chinese STORI-30 five subscales with these two convergent measurements. That was, Stage 1 had negative and significant correlations with RAS-C (r = -0.55, p < 0.01) and C-SWEMWBS (r = -0.45, p < 0.01); other recovery stages had positive and significant correlations with those two measures (r = 0.22 to 0.76). In general, the higher recovery stages the higher level of positive correlations. For divergent validity, stage allocation had negative and moderate correlation with SSS-S (r = -0.35, p < 0.01). Stage 1 had positive and moderate correlation (r = 0.45, p < 0.01) whereas Stage 5 had negative and moderate correlation (r = -0.40, p < 0.01). The higher recovery stages the greater level of negative correlations with self-stigma.

**Table 3:** Correlations of three factors with convergent and divergent measures (N = 113)

	Convergent and divergent measures		
Chinese STORI-30	RAS – C total score	C-WEMWBS total score	SSS-S total score
Factor 1	0.56**	0.34**	-0.19*
Factor 2	0.76**	0.65**	-0.34**
Factor 3	-0.63**	-0.56**	0.49**

*p < 0.05; **p < 0.01

Chinese STORI-30 = Chinese version of Stage of Recovery Instrument-30; RAS-C = Recovery Assessment Scale – Chinese version; C-WEMWBS = Chinese version of the Short Warwick-Edinburgh Mental Well-being Scale; SSS-S = Self-Stigma Scale – short form

**Table 4:** Correlations of Chinese STORI-30 stage allocation and subscales with convergent and divergent measures (N = 113)

	Convergent and divergent measures		
Chinese STORI-30	RAS – C total score	C-WEMWBS total score	SSS-S total score
Stage allocation	0.61**	0.60**	-0.35**
Stage 1	-0.55**	-0.45**	0.45**
Stage 2	0.41**	0.22*	0.23
Stage 3	0.63**	0.45**	-0.22*
Stage 4	0.76**	0.58**	-0.35**
Stage 5	0.75**	0.72**	-0.40**

*p < 0.05; **p < 0.01

Chinese STORI-30 = Chinese version of Stage of Recovery Instrument-30; RAS-C = Recovery Assessment Scale – Chinese version; C-WEMWBS = Chinese version of the Short Warwick-Edinburgh Mental Well-being Scale; SSS-S = Self-Stigma Scale – short form

### Criterion validity

A total of 37 (32.7%) caseworkers rated on their clients’ recovery stage. On average, participants received services from their caseworkers for 10.6 weeks (SD = 10.0). A fair level of agreement was found between caseworkers’ and participants’ rating on recovery stage (K = 0.35, p < 0.01). Considering the high intercorrelations between adjacent recovery stages, further analysis was performed. Collapsing Stage 3, 4 & 5 into one stage while keeping Stage 1 & 2 as separate stages, agreement level increased to moderate level (K = 0.44, p < 0.001).

### Test-retest reliability

Total 39 (34.5%) participants agreed to complete Chinese STORI-30 again and the time interval ranged from two to four weeks. Average measure ICC of stage allocation was 0.96 and the five subscales scoring were ranged from 0.81 to 0.85, reflecting a high degree of test-retest reliability.

### Descriptive statistics

Using parametric testing (independent t-test, One-Way ANOVA and Pearson correlation), no significant difference was found in Chinese STORI-30 stage between groups based on gender, age, education, diagnosis and employment status. There was no significant correlation between recovery stage and years from onset of mental illness or length of last hospitalisation.

## Discussion

The strengths and limitations of present study are worthy of note. In view of content validation process, it involved a judiciously selected expert panel and explicit quantifying methods. Satisfactory results of item- and scale-level CVIs indicate adequate relevance and representativeness to the construct of recovery stage [40]. Besides, satisfactory agreement level among service users further strengthen the evidence of feasibility and utility in clinical settings.

The EFA of this study revealed an ambiguous three-factor solution and the factor loading pattern is comparable to the four-factor structure of original STORI-30 study. Commonalities were shown in both studies, for example, Stage 1 remained as a discrete factor; Stage 2 merged with certain items of Stage 3 and Stage 4. On the other hand, we noted that in this study, Stage 3 grossly dispersed into two factors. This might be explained by the fact that this recovery stage conceptually correlates with the Stage 2 and Stage 4, therefore, it is hard to be discretely distinguished as an independent factor. In fact, the three-factor structure derived from our EFA complements similar pattern of three-cluster solution obtained by hierarchical cluster analysis in the development of STORI: cluster 1 contained all Stage 1 items; cluster 2 contained items of Stage 2, 3 and 4; and cluster 3 contained items of Stage 4 and 5.

Despite this study failed to prove an expected five-factor structure, it would be explained by the complexity of recovery model. Recovery components across the five stages might actually cultivate in a non-linear or continual spiral pattern instead of a lockstep pattern [41]. For instance, a patient might acquire significant sense of hope but a low level of responsibility-taking before moving forward to preparation stage. Complexity of model imposes challenges to distinguish recovery stages quantitatively, and hence it merits attention for re-examination of Chinese STORI-30 that whether or not a five-stage model would be confirmed or abandoned. Nevertheless, structural validity evidence suggested high interrelatedness under each recovery stage, and the sequential nature of five stages was supported as described theoretically.

Consistent with the original studies of STORI-30 and STORI, positive correlations pattern was found between Chinese STORI-30 and measurements of psychological recovery and mental well-being. It is no surprise that people at higher recovery stages acquired greater recovery components. And hence, Factor 2 which contained items of later recovery stages, was highly correlated with these scales. Factor 1 showed weaker correlations because it contained items of earlier recovery stages. Furthermore, this study took initiation to seek new evidence of divergent validity. Factor 3 which contained all items of Stage 1 Moratorium, demonstrated a significant and positive correlation with self-stigma scale. This would be justified by patients’ wretched sense of frustration and rooted illness role, which might be hardly eliminated when being a member of devalued mental illness group in the society. Besides, a fair agreement level between participants’ and caseworkers’ rating on recovery stage was found. It might be contributed to the discrepancy of conceptualisation among stakeholders, and thus additional descriptions would possibly help them having a better understanding about recovery stages. Nonetheless, service users believed the STORI-30 shall be utilised by clinicians for taking consumers’ perspective while implementing recovery services [20].

### Study limitations

First, it was extraordinarily challenging to recruit participants under pandemic, and unfortunately the general rule of five to ten respondents for each testing item was failed to achieve [23]. Inadequate sample size of current study might lead to problems manifesting in factor structure results, and hinder the data generalisation to a larger population [42]. Thus, a larger sample size would overcome sampling errors and create a more reliable factorial structure solution.

Furthermore, a skewed spread of stage allocation to high recovery stages was noted. This might be a sampling error explained by recruitment from merely outpatient and community services. Majority of participants were actively receiving rehabilitation services or living at half-way house, they were expected to be high functioning and in a later recovery stages. In fact, this study made an effort to recruit a wide span of participants from diversified service units such as residential services, vocational rehabilitation units, and integrative community mental health centers. To further enhance quality of data sampling, participants who are with severe dysfunction and likely positioning at early recovery stages, shall be recruited from long-stay care home or inpatients of chronic wards at hospitals on the prerequisite of stable mental state.

## Conclusion

This study made the first attempt to translate STORI-30 from original English to traditional Chinese, and validate it on a group of adults with SMI in Hong Kong. Local mental health experts attained agreement on the content validity, and feedbacks collected from consumers affirmed feasibility and utility. More importantly, empirical evidence of psychometric properties supported the notion that Chinese STORI-30 is a valid and reliable instrument of consumer-oriented recovery stage. The five stages of recovery presented in ordinal sequential nature as described by the theoretical model, and each recovery stage demonstrated high internal consistency. Also, Chinese STORI-30 was congruent with other recovery and mental well-being measurements, but against self-stigma construct. Additionally, test-retest reliability was confirmed that the scale remained stable over a period of time. Nevertheless, empirical data suggested potential overlapping between adjacent recovery stages, and thus it remains uncertain whether or not Chinese STORI-30 discriminates consumers into five recovery stages.

A validated instrument of recovery stage significantly serves as a useful reference to both mental health workers and consumers. Not only does Chinese STORI-30 accommodate individuals’ personal recovery experiences but it also facilitates service providers for developing mental health interventions tapping on the need of each recovery stage. In the near future, there is considerable research gap that validation studies would be worthwhile with a larger sample size, in order to re-examine the underlying construct of Chinese STORI-30, as well as emergent new evidence to the theoretical framework of recovery stages.

## Data Availability

The datasets analysed during the current study are included in this published article.
